# Association of tissue lineage and gene expression: conservatively and differentially expressed genes define common and special functions of tissues

**DOI:** 10.1186/1471-2105-11-S11-S1

**Published:** 2010-12-14

**Authors:** Yao Yu, Tao Xu, Yongtao Yu, Pei Hao, Xuan Li

**Affiliations:** 1 Key Lab of Systems Biology/Key Laboratory of Synthetic Biology, Shanghai Institutes for Biological Sciences, Chinese Academy of Sciences, Shanghai 200031, China; 2 Graduate School of the Chinese Academy of Sciences, Shanghai 200031, PR China; 3 College of life science and biotechnology, Shanghai Jiaotong University, Shanghai 200240, China; 4 Shanghai Center for Bioinformation Technology, 100 Qinzhou Road, Shanghai 200235, China

## Abstract

**Background:**

Embryogenesis is the process by which the embryo is formed, develops, and establishes developmental hierarchies of tissues. The recent advance in microarray technology made it possible to investigate the tissue specific patterns of gene expression and their relationship with tissue lineages. This study is focused on how tissue specific functions, tissue lineage, and cell differentiation are correlated, which is essential to understand embryonic development and organism complexity.

**Results:**

We performed individual gene and gene set based analysis on multiple tissue expression data, in association with the classic topology of mammalian fate maps of embryogenesis. For each sub-group of tissues on the fate map, conservatively, differentially and correlatively expressed genes or gene sets were identified. Tissue distance was found to correlate with gene expression divergence. Tissues of the ectoderm or mesoderm origins from the same segments on the fate map shared more similar expression pattern than those from different origins. Conservatively expressed genes or gene sets define common functions in a tissue group and are related to tissue specific diseases, which is supported by results from Gene Ontology and KEGG pathway analysis. Gene expression divergence is larger in certain human tissues than in the mouse homologous tissues.

**Conclusion:**

The results from tissue lineage and gene expression analysis indicate that common function features of neighbor tissue groups were defined by the conservatively expressed genes and were related to tissue specific diseases, and differentially expressed genes contribute to the functional divergence of tissues. The difference of gene expression divergence in human and mouse homologous tissues reflected the organism complexity, i.e. distinct neural development levels and different body sizes.

## Background

Investigating the expression divergence of multiple tissues is important to understand the organism complexity [1]. Currently, the advance in microarray technology has provided a huge amount of quantitative data of tissue housekeeping and tissue specific gene expression in many species [2]. Some studies have focused on the relationship among tissue specificity, expression conservation, expression level, and sequence conservation [3,4]. For example, the evolutionary rate of expression divergence and that of coding sequence divergence were found to be weakly, but significantly positively correlated. Others paid attention to housekeeping, tissue-specific gene and tissue-specific transcriptional regulation for revealing molecular fundamental of tissue function and their evolutionary characters [5-7]. Results from these studies showed that different tissues shared a large number of ubiquitously expressed genes and the tissue specific genes are more likely to reveal tissue-specific function. A gene set (Gene Ontology - GO) based analysis proposed a “tissue-driven” hypothesis which describes the relationship between the stabilizing constraints on tissue-specific gene expression and individual GO categories [8].

Embryogenesis is the process by which the embryo is formed, develops, and establishes developmental hierarchies of tissues. It describes the developmental history from the single-celled zygote to the multi-celled adult [9,10]. The construction of *Caenorhabditis elegans* cell fate map traces the sequence of cell division, migration, and apoptosis of each of the 671 cells [11]. Fate maps of mammals [12] were proved necessary to construct developmental hierarchies of tissues. For example, during the early stages of embryonic development, the brain starts to form in three distinct segments: the prosencephalon, mesencephalon, and rhombencephalon. The cerebellum later develops from the rhombencephalon, which is the caudal portion of the embryonic brain [13]. The gonads which in males are the testes and in females are the ovaries develop from the intermediate mesoderm, which is found between the paraxial mesoderm and the lateral plate [7,14]. It is also from the intermediate mesoderm the mammalian kidney develops.

To investigate the gene expression similarity and specificity of tissues in association with the fate map of embryogenesis is of great important to understand how tissue specific functions, tissue lineage, and cell differentiation are correlated in the context of embryonic development and organism complexity. To predict the potential disorders of a tissue from its expression profile might lead to early diagnosis of certain diseases and identification of congenital defects in clinics. In the current study, by combining gene expression profiling from multiple tissues with the mammalian fate maps of embryogenesis, we investigated tissue lineage and cell differentiation through mammalian development at the molecular level. We introduced individual gene and gene set based approaches [15] for evaluating tissue expression similarity and divergence. To provide a valuable resource for the in-depth understanding of tissue development and tissue specific functions, we created a gene and gene set expression map along the paths of mammalian embryogenesis. Herein, we present our results on the conservatively, differentially and correlatively expressed genes/gene sets for each sub-group of tissues on the fate map. By Gene Ontology and KEGG (Kyoto Encyclopedia of Genes and Genomes) pathway analysis, we further define the functions of each gene group. In addition, by comparing the data from human and mouse tissues, we investigate the inter-species divergence of expression profile in tissues. Our results provide valuable insight into how developmental hierarchies and gene expression are coordinated, and help understand tissue specific functions in relation to cell differentiation features.

## Methods

### Data description and preprocess

The human (HG-U133A and GNFIH) and mouse (GNF1M) Affymetrix microarray data (Affymetrix, Snata Clara, CA) [2] were retrieved from Gene Expression Omnibus (GEO) of National Center for Biotechnology Information (NCBI) [16] (Human: GDS594 and GDS596; Mouse: GDS 592). The data resource contains the expression data from 73 human tissues (GDS594/GDS596) and 69 mouse tissues (GDS592). Only common homology tissues from human and mouse were considered in the following analysis (24 orthologous adult tissue listed in Additional file 1). And for those biological replicates we assigned the average expression value as the signal. After mapping probes to the microarray chip platform annotation files, the final datasets include 14746 human genes and 13048 mouse genes. The homology gene pairs’ information in human and mouse was gained from NCBI (http://www.nicbi.nlm.gov/HomoloGene). After extracting the unique hit homology pairs, we identified 5055 orthologous gene pairs have expression data in both human and mouse. We used packages from BioConductor (http://www.bioconductor.org/) for functional annotation of those genes. Package ‘org.Hs.eg.db’ and ‘org.Mm.eg.db’ (version 2.2.11) were used for Gene Ontology (GO) mapping of human and mouse genes respectively. Package ‘KEGG.db’ (version 2.2.11) was used for pathway mapping of genes. Only these GO modules with over five genes on the chip will be used in the following analysis.

### Estimation of gene expression divergence in tissues

A p-rank method [17] was performed to evaluate the gene expression distance between two tissues. To obtain the p-rank value of gene expression, a gene’s expression signal value was first ranked among all genes in each tissue, and then divided by the number of genes *n*.

Let *p_i,t1_* and *p_i,t2_* indicate the p-rank values of gene *i* in tissues *t1* and *t2*. We calculated the gene divergence *E_i_* for every gene between each two tissues on the fate map as:

*E_i_* = |*p_i,t_*_1_ – *p_i,t_*_2_|

In each pair of tissues, those genes with divergence in the lowest 5% of all gene divergence values were classified as the conservatively expressed genes, which have the least expression divergence between two tissues. Furthermore, those genes which were expressed as the top 5% in a group of tissues or in all the tissues were considered as ubiquitously conservatively expressed genes in corresponding tissue group.

### Gene set based measurement for inter tissue expression similarity

Gene Ontology (GO) [18] provides a controlled vocabulary of terms for describing gene product characteristics and gene product annotation data. Consider a set of n genes in one Gene Ontology module; three kinds of measurements were defined for characterizing the profiling of this GO among the tissues. If expression distance was significantly small for a given GO module in a pair of tissues, the expression level of this module was considered conservative in the tissue pair. If a significant *p* value was obtained in the Kolmogorov–Smirnov test (KS test) [19] for the expression of a given GO module in a pair of tissues, the expression distribution of this module was referred to as a divergent pattern in these tissues. If Pearson correlation coefficient was significant for a given GO module in a pair of tissues, their expression relationship was referred to as a linear correlated pattern [20].

#### Tissue expression distance

Let *S_i,g,t_* denote the (log2 transformed) expression levels of gene *i* of GO *g* in tissue *t*. The expression distance of GO *g* in tissue *t1* and tissue *t2* is calculated as [4]:

while *n* is the number of gene in GO *g.*

The *D* values in all tissue pairs in human and mouse approximately follow a normal distribution (see in Additional file 2). Based on the cumulative distribution function of the normal distribution, a *p* value was calculated to represent the significance of the expression similarity of GO *g* in tissue *t1* and tissue *t2:*

*µ* for the mean of *D* values and *σ* for the standard deviation of *D* values.

For a sub-group of tissues in the fate map’s branch, the intro group expression similarity of GO *g* is estimated using an integrated p value [21]. Since *D ~ N*(*µ,σ*^2^)*, D_sub_*(the mean value of *D* values in a sub-group of tissues) should follow *D_sub_~ N*(*µ,σ*^2^*/ N*)*, N* for the sample size in this group. Thus the integrated p value could be calculated as:

*P_g,t–sub_* = Pr(*X ≤ x*) = *ϕ_µ,σ_*_^2^*/ N*_(*x*), *x* = *D*_*g,t*1–*t*2_

The GO module with a small p value or integrated p value was considered as conserved GO module in a pair or a group of tissues.

#### Tissue expression difference

To identify differentially expressed GO modules in a pair of tissues, a nonparametric KS test was performed [22]. The p-rank values of the genes in certain GO module from a pair of tissues were used. A *p* value (denoted as *p_g,t1-t2_*) less than 0.05 denoted that the difference of expression status of this GO module in two tissues should be considered as statistically significant.

For a group of tissues, the *p* values from the KS test in each pair of tissues from that group were integrated. The *p* values *p_g,ti-tj_* were transformed to *Z_g,ti-tj_* with quantile function of normal distribution. Then *Z* score for a sub-group of tissues (*Z_sub_*) was summarized from *Z_g,ti-tj_* with the function:

, n for the number of pair wise p values in this group

If these GO modules were not signature modules of a group of tissues, the *p_g,ti-tj_* would follow uniform distribution. If *p_g,ti-tj_* followed uniform distribution, *Z_g,ti-tj_* would follow norm distribution. As a result, *Z* score would also follow norm distribution. A significant small value of *Z* comparing to normal distribution corresponded to the significantly being perturbed of the GO modules under these tissues.

#### Tissue expression correlation

The expression correlation of GO *m* in a pair of tissues *t1* and *t2* was defined as the Pearson’s correlation coefficient [23] of the profiles of genes in GO *m* in tissue *t1* and in tissue *t2*:

, while *n* is the number of gene in GO *m*.

A high *r* indicates a high similarity of the expression profiles of the GO module in two tissues, thus this GO module was considered as correlated expressed GO module in tissues. The threshold for *r* between two tissues was set to be *r* > 0.9, while the threshold for *r* in a group of tissues was set to be *r_mean_* > 0.9.

### Gene enrichment analysis

For all the genes defined as tissue conserved ones, we made gene module enrichment using in GO modules and KEGG pathways. The enrichment significance of tissue conserved genes was calculated in a hyper genomic distribution model. Let *k* be the number of sequences corresponding to genes of interest (i.e. conservatively expressed genes) in certain GO module (or KEGG pathway). The total number of sequences in genes of interest is *n.* Given the total size of genes in the libraries (*m*) and in all tissues (*N*), the probability of observing *k* or more sequences for gene *g* in liver can be calculated by the formula:

The Bonferroni correction was used to adjust the p-values for liver enrichment gene identification based on the hyper-geometric distribution [21]. When performing n tests, with each of them being significant with probability β, the probability that at least one of them comes out significant should be less than n*β. The calculation was conducted in R platform with ‘stats’ package (http://www.r-project.org).

### Supplementary files

The analysis workflow was presented in Additional file 3. All the supplementary files were available at our website: http://omics.biosino.org:14000/kweb/tissue_expression/

## Results

### Estimation of gene expression divergence in tissues

**Figure 1:**
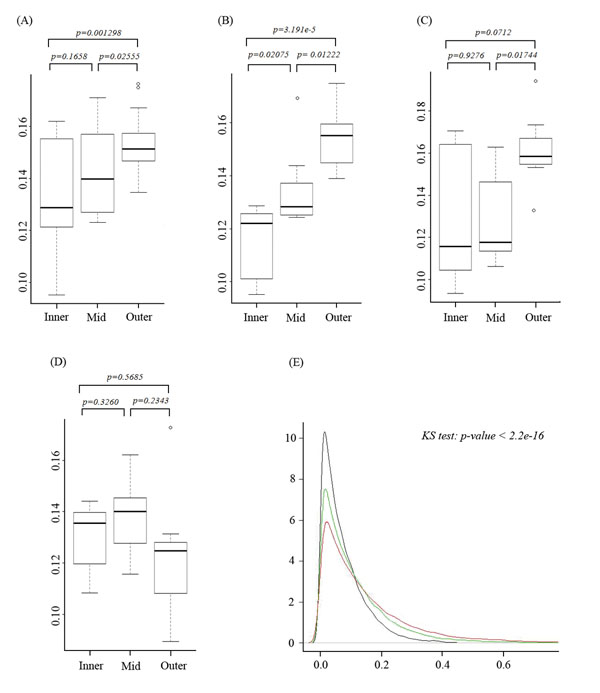
The distribution of gene expression distance in tissue pairs: (A) for all tissues considered in this study, (B) for ectoderm tissues, (C) for endoderm tissues, and (D) for mesoderm tissues. ‘Inner’ represents the group of neighbor tissues, defined as tissues belong to the same super-node (red rectangle in Figure. 2). ‘Mid’ represents the group of tissue pairs from the segments of ectoderm, endoderm and mesoderm, while not in the same ‘inner’ group. ‘Outer’ represents the group of tissue pairs from different segments. Student's t-tests were performed on each data pairs of these groups from (A) to (D). (E) The gene expression divergence between amygdale and other tissues. The black curve represents the density of divergence between amygdale and prefrontal cortex. The green curve represents the density of the mean divergence of amygdale and other neural tissues. Red curve represents the mean divergence density between amygdale and other somatic tissues. KS test was performed to test the significance of the difference of these curves.

Based on p-rank transferred value (original data in Additional file 4), individual gene expression divergence (see in Methods) in different tissue pairs was estimated. Our results showed the expression divergence varies greatly in different tissue pairs. Generally, the divergence of the neighbor tissues on the fate map is less significant than randomly picked pairs (Figure 1A). In addition, the degree of the difference in gene expression profile from two tissues is positively correlated with their distance on the fate map. According to both t-test and KS test, the gene expression distance within neighbor tissues was significantly smaller than that from relatively distant tissues on the fate map. This phenomenon is especially clear in the neural system from ectoderm (Figure 1B) and in tissues from endoderm (Figure 1C), e.g. the distribution of gene expression distances between amygdale and other tissues (Figure 1E). In the comparison pair of amygdale and its closest neighbor prefrontal cortex, more genes were identified as conservatively expressed ones than in other tissue comparison pairs. Furthermore, the lowest peak from the pairs of amygdale and other none neural tissue represented a higher expression divergence in those tissues. However, in the mesoderm tissues, the trend of the decreasing of gene expression conservation along with tissue distance on the fate map was not that significant (Figure 1D). This result suggested that through the process of embryogenesis, tissues inherit some imprinting from their ancestor. However, due to the function divergence and developing timing difference less was inherited in mesoderm tissues than in other segments. This phenomenon was consistently observed in both human and mouse (see in Additional file 4 and Additional file 5).

Certain individual genes are extremely conservatively expressed in a group of tissues from one branch of the fate map. These genes might always be closely related to the specific function of those tissues. For example, in human, gene *CALM2*, which is well known as the important phosphorylase kinase in calcium signaling pathway [24], vital for transduction of nerve stimulus -a basic function for the nerve cells, was conservatively expressed through all tissues derived from neural tube (Additional file 4). Interestingly, in mouse, the kinase-coding gene *Camk2g* with similar function was conservatively expressed in these tissues, too. From gene set enrichment analysis, we found that conservatively expressed gene sets in the tissue group from the same ancient segments in embryogenesis define certain common functions in the tissue group. Such phenomenon was quite explicit when we examined the tissue group of high order on the fate map, such as the group of tissues developed from mesoderm, ectoderm and endoderm, etc. For example, the GO modules for fundamental biological processes, i.e. nucleic acid binding (GO:0003676), structural constituent of ribosome (GO:0003735), and structural constituent of ribosome (GO:0003691), were identified to be extremely conserved in all the tissues (Additional file 6 for human and Additional file 7 for mouse) in human. Another example was the relationship between tissue-conserved genes and diseases. Those genes conservatively expressed in the cerebellum and olfactory bulb were significantly enriched in Prion diseases related pathway (HSA05020, Additional file 6).

### Tissues gene expression distance in view of Gene Ontology

**Figure 2:**
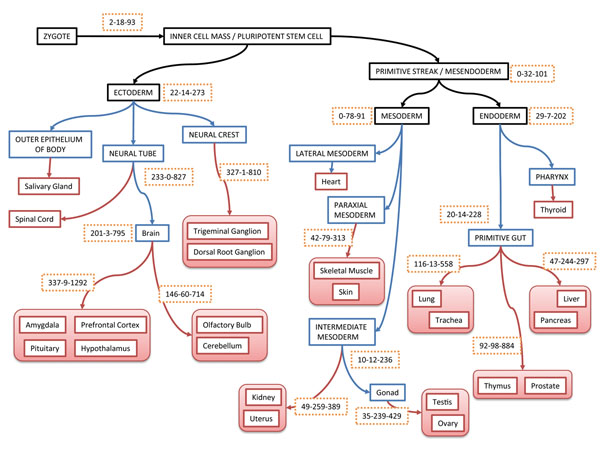
The embryogenesis fate map of 24 adult tissues considered in this study and gene set based tissue divergence analysis results. The topology of embryo fate map was constructed mainly based on *“Developmental Biology”*[30]. The red nodes on the tree represent the adult tissues used in microarray experiment. The blue nodes represent the middle stages in developing process. The black nodes represent the primitive organizes of embryo. The numbers in the frame with dotted edges represent the numbers of identified GO modules according to three kinds of measurement: conservative expressed gene sets according to expression distance, differentially expressed gene sets according to KS test, and correlatively gene sets according to Pearson’s correlation.

We calculated the expression distance of GO categories in each pair of tissues. Conservatively expressed gene set was defined as the one with similar expression level in a pair (group) of tissues. Accordingly, conserved GO modules were identified in those sub-groups of tissues on the fate map. Figure. 2 illustrated the number of conserved GO modules on each node of the fate map. Generally, the number of conserved GO modules in sub-groups of tissues was decreasing along with the process of development from zygote to adult tissues. There is no conserved GO module detected, considering all tissues from mesendoderm and zygote. This is consistent with the fact that tissue developing process is reflected by expression divergence growing at molecular level. In different developing branches, the growing of gene set divergence has large variance. For instance, 201 GO modules had been identified as conservative modules in brain, while 20 GO modules had been identified as conservative ones in primitive gut. These conservative GO modules in brain concentrate on such functions as the regulation of G-protein coupled receptor protein signaling pathway (GO:0045744), as well as certain cell cycling related housekeeping functions (Additional file 8). The conservative GO modules in gonad (testis and ovary) contain growth hormone receptor signaling pathway (GO:0060396) and response to growth hormone stimulus (GO:0060416). All these conservative GO modules in different node on the fate map have close relationship with the common functions of the sub-group of adult tissues.

KS tests were performed to detect significantly differentially expressed GO modules, of which the distribution of the gene expression levels varies greatly in different tissues. Tissues from mesoderm and endoderm segments contain more differentially expressed GO modules found by pair wise comparisons of these tissues (Figure 2). The GO modules for synaptic transmission (GO:0007268, GO:0019226) and transmission of nerve impulse (GO:0045202) were identified as the top three significantly differentially expressed ones in brain, while in the sub-group of brain (prefrontal cortex, amygdale, pituitary, hypothalamus) the expression profiles of these modules remain stable (Additional file 9). The cerebellum has an important function in the integration of sensory perception, coordination and motor control. In order to coordinate motor control and response to stimulation, genes in these modules are actively expressed in this region to link the cerebellum with the cerebral motor cortex (which sends information to the muscles causing them to move) and the spinocerebellar tract (which gives proprioceptive feedback on the position of the body in space) [25]. Besides, the significantly differentially expressed GO modules in ectoderm contain function modules for immune system (GO:0002376, GO:0006955), response to defense (GO:0006952), response to stimulus (GO:0050896), etc. Thus, the differentially expressed GO modules contribute to the function divergence of tissues.

Using Pearson’s correlation coefficient, correlated expressed GO modules were identified. The expression of genes in these modules simultaneously up-regulated or down-regulated (or remain same) among different tissues (Additional file 10). Neural related tissues contain more correlated expressed GO modules than the tissues developed from mesoderm and endoderm. There were 827 and 810 highly correlated (correlation coefficient > 0.9) GO modules in neural tube and neural crest, respectively. However, the correlated expressed modules in intermediate mesoderm and primitive gut were 236 and 228, respectively. These results suggested that the gene expression in non-neural tissues is more divergent than in neural related tissues. Most of the correlated expression genes of neural tissues were enriched into such pathways like signal peptide processing (GO:0006465), G-protein signaling pathway (GO:0007189, GO:0010578 and GO:0010579), BMP signaling pathway regulation (GO:0030510). Comparatively the expression of these GO modules in endoderm and mesoderm tissues showed no significant correlation.

### Tissues gene expression in human and mouse

Gene set (GO module) based methods for estimating the expression divergence in tissues on the fate map were used to process the mouse data (Additional file 11, 12, 13). Compared with the results from the human data, the trends of tissue divergence are similar in both species (Table 1). Neural related tissues developed from ectoderm were the most conservative tissue group on the fate map. In mouse, the GO modules expression distances in this group of tissues were significantly shorter than those from endoderm and mesoderm tissues. The KS tests showed that no significant differentially expressed GO module could be identified in ectoderm tissues. The most different tissue sub-group on the fate map in expression level is gonad (consisting of testis and ovary; 228 GO modules differentially expressed by KS test), which is consistent with the results in human (239 differentially expressed GO modules). There were more correlated expressed GO modules in ectoderm tissues than in other tissues on the fate map.

**Table 1:** Gene ontology based tissue expression divergence.

Tissue Node	Human (4207 GO modules)			Mouse (2321 GO modules)			Annotation
	GO Distance	KS.test	Correlation	GO Distance	KS.test	Correlation	
Zygote	2	18	93	2	3	94	pluripotent stem cell
Mesendoderm	0	32	101	1	12	102	endoderm & mesoderm
Endoderm	29	7	202	1	29	109	endoderm tissues
PrimitiveGut	20	14	228	3	26	122	primitive gut 1 & 2 & 3
PrimitiveGut3	92	98	884	64	78	535	thymus & prostate
PrimitiveGut2	47	244	297	7	124	115	pancreas & liver
PrimitiveGut1	116	13	558	180	4	1399	trachea & lung
Mesoderm	0	78	91	20	15	222	mesoderm tissues
ParaxialMesoderm	42	79	313	108	18	722	skin & skeletal muscle
IntermediateMesoderm	10	120	236	30	47	255	intermediate
KU	49	259	389	91	30	600	uterus & kidney
Gonad	35	239	429	33	228	235	testis & ovary
Ectoderm	22	14	273	111	0	703	ectoderm tissues
NeuralCrest	327	1	810	437	0	2106	neural crest
NeuralTube	233	0	827	241	0	1587	neural tube
Brain	201	3	795	216	0	1524	brain 1 & 2
Brain2	146	60	714	368	0	1728	cerebellum & olfactory bulb
Brain1	337	9	1292	224	0	1491	prefrontalcortex & amygdala & pituitary & hypothalamus

The thresholds for these measurements are: GO Distance: p < 0.05 (or integrated p < 0.05); KS test: p < 0.05 (or integrated p < 0.05); Correlation: r > 0.9.

**Figure 3:**
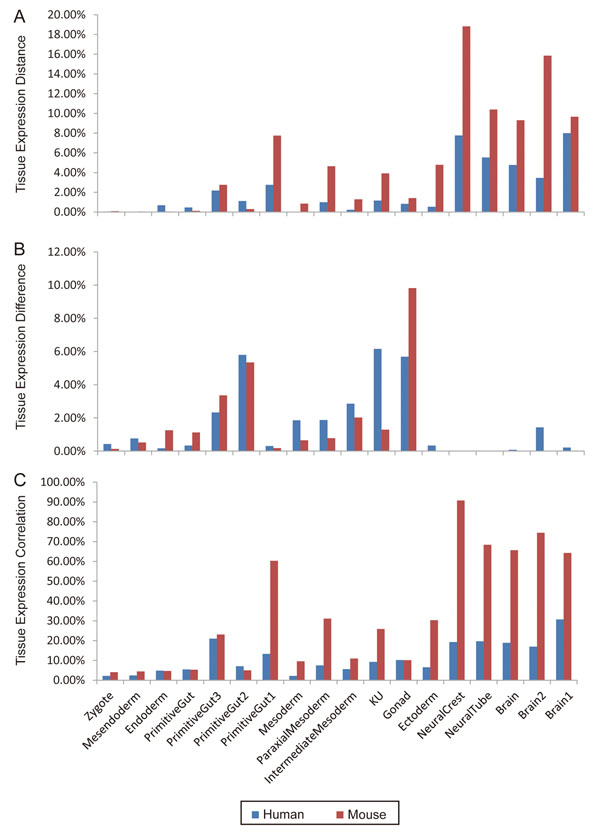
Comparisons of conservatively expressed GO modules (A), differentially expressed GO modules (B), and correlatively expressed GO modules (C) between human and mouse tissues. Red bars denote the mouse tissues, and blue bars the human tissues. Each bar height represents the percentage of significant GO modules (p<0.05) out of the total number of GO modules analyzed from mouse or human. The names of tissue group nodes on the X axis are consistent with those used in Table 1.

The comparison of the numbers of conservatively and differentially expressed GO modules in human and mouse tissues was illustrated in Figure 3. Compared with the common size adjusted data, the mouse tissues contain more conserved GO modules and less differentially expressed ones than the corresponding human tissues. Also there exists significant difference in gene set expression of individual tissues. Uterus and kidney (noted as KU) in mouse have more similar expression profile than their homologous tissues in human. According to expression distance in KU, there were more conserved GO modules in mouse (91 GO modules) than that in human (49 GO modules), while according to KS test there were less differential expressed modules in mouse (30 GO modules) than that in human (259 GO modules). In addition, there are much more highly correlated expressed GO modules in tissues in mouse than in human, especially in tissues from ectoderm. Regardless of the fact that there are fewer mouse genes than human genes on chip, which might create bias for the analysis, our results suggested a less divergence in mouse tissues at molecular level than in human tissues. This phenomenon is more pronounced in the nervous system and the paraxial mesoderm. According to Alexander E., et al [26], the prominent human–mouse divergence in neural system might reflect both the increase in organism complexity and the transition to the qualitatively higher complexity level: social organization. Besides, the higher cell differentiation level in paraxial mesoderm (such as skeletal muscle) might be due to the larger body size.

## Discussion

In this study, we mapped human and mouse tissues onto the mammalian fate map of embryogenesis and further analyzed the expression conservation of tissues with individual genes and gene set (Gene Ontology) based methods. We tested the hypothesis the tissues from the same branch on the fate map shared more similarity in gene expression profile with each other than their remote neighbors. Our results showed this hypothesis was true in ectoderm and mesoderm, where tissues from the same segments on the fate map share more similar expression patterns than those from different origins of embryogenesis. However, this assumption is not true in endoderm tissues. The molecular basis for such phenomenon may be related to gene imprinting that tissues inherit from their ancestors throughout the development process. The imprinting might involve DNA methylation, histone modification, and other epigenetic mechanisms that lead to the conservation of gene expression in neighbor tissues and divergence in distant segments. In addition, the expression divergence of genes or gene sets varied greatly in different sub-group of neighbor tissues on the fate map. Neural tissues developed from ectoderm were the most conserved sub-group. The expression pattern of genes in these tissues is more conservative to each other: more conserved individual genes, more conserved GO modules, less differentially expressed GO modules and more significantly correlated GO modules, compared with tissues developed from mesendoderm [8]. Notably, tissues developed from endoderm appear to have a large number of differentially expressed genes, which might be related to the large functional variation of these tissues and the long developing time in embryo.

In most tissues, tissue-specific functions are reflected in the stably expressed genes and GO categories. For example, genes from ubiquitin mediated proteolysis and ribosome pathway show universal conservation in all tissues, and their expression levels in all sub-groups of tissues remain stable. Different tissue groups of the same origin have identical expression conserved genes. *CALM* of human genome or *Camk2g* of mouse genome are conservatively expressed in brain and other tissues developed from neural tube, whereas neither these genes nor the calcium signaling pathway is detected in the conservation and enrichment analysis of mesendoderm tissues. The construction of tissue specificity is more likely to be governed by those gene sets with specific molecular functions. Prion disease pathway was identified as conservative gene enriched pathway in the cerebellum and olfactory bulb. The *Prnp* (prion protein) was one of the conservative genes in this pathway, which also plays the role as the pathological agent in transmissible spongiform encephalopathies such as bovine spongiform encephalopathy in cattle, scrapie in sheep, and Creutzfeldt–Jakob disease in humans [27]. This gene is considered a potential biomarker for early diagnosis of prion disease, as the *Prnp-mutant* over-expression changes the structure of its globular domain and induces a lethal spongiform encephalopathy [28].

The timeline difference of tissue development and maturity might account for the phenomenon that there is larger divergence of gene expression in tissues from ectoderm than in tissues from mesoderm and endoderm. By week 20 of gestation, fetal kidneys, urinary tract, and digestive system start functioning by producing urine and meconium. The neural system, however, reaches maturity much later. In Rhombic lip, a primary region that produces the neurons to make up the cerebellum, neurons migrate to the external granular layer until embryonic week 27. The later differentiation of neural tissues might lead to less conservative gene expression pattern at molecular level. Another important factor that might affect gene expression is microRNAs, which have been shown to regulate target gene expression in tissues at different developmental stages [29]. By cross-species comparison, Cui et al. found that the expression variation of miRNA targets is significantly lower than that of other genes, which implies miRNAs function as constrain to reduce target gene expression variation. It remains interesting to see whether miRNAs play similar roles between mammalian species, i.e. mouse and human.

By comparison of gene and gene set expressions between human and mouse homologous tissues, we observed some major differences in certain tissues’ gene expression patterns, e.g. smaller gene expression divergence of neural tissue group in mouse (denoted as more conservative GO modules in mouse ectoderm tissues), and more conservative expression of paraxial mesoderm in mouse. Although the trend of “tissue-driven” (lower divergence of gene expression in more stringent tissues and higher divergence in more relaxed tissues) in gene expression remains unchanged between human and mouse, the difference in gene expression divergence between certain human and mouse tissues is mainly attributed to the highly developed human neural system supporting intelligence and the large difference in their body sizes.

## Conclusions

The recent advance in microarray technology made it available to use a large amount of tissue expression data for the investigation of the tissue specific expression pattern in association with tissue lineage and development. This study was designed to learn how tissue specific functions, tissue lineage, and cell differentiation are correlated, which is essential to understand embryonic development and organism complexity. By performing individual gene and gene set based analysis on multiple tissues expression data in association with the classic topology of mammalian fate maps of embryogenesis, we identified conservatively, differentially and correlatively expressed genes or gene sets for each sub-group tissue on the fate map. The results from tissue gene expression, and Gene Ontology and KEGG pathway analyses indicate that common function features of neighbor tissue groups were defined by the conservatively expressed genes and were related to tissue specific diseases, and differentially expressed genes contributed to the functional divergence of tissues. The difference of gene expression divergence in human and mouse homologous tissues reflected the organism complexity, i.e. distinct neural development levels and different body sizes.

## List of abbreviations used

GO: Gene Ontology; KEGG: Kyoto Encyclopedia of Genes and Genomes; NCBI: National Center for Biotechnology Information; KS test: Kolmogorov–Smirnov test.

## Competing interests

The authors declare that they have no competing interests

## Authors' contributions

YY and TX performed design and statistical analyses for the study, and drafted the manuscript. YY participated in the design of the study and provided guidance. PH and XL conceived the study, and finalized the organization and contents of the manuscript. All authors approved the final manuscript.

## Supplementary Material

Additional file 1 Tissue list and GSM summary Click here for file

Additional file 2 Histogram of Human GO distance Click here for file

Additional file 3 Analysis workflow Click here for file

Additional file 4 Human gene tissue expression distance Click here for file

Additional file 5 Mouse gene tissue expression distance Click here for file

Additional file 6 Human tissue conservatively expression gene enrichment analysis Click here for file

Additional file 7 Mouse tissue conservatively expression gene enrichment analysis Click here for file

Additional file 8 Human GO modules tissue expression distance Click here for file

Additional file 9 Human GO modules tissue expression difference test Click here for file

Additional file 10 Human GO modules tissue expression correlation Click here for file

Additional file 11 Mouse GO modules tissue expression distance Click here for file

Additional file 12 Mouse GO modules tissue expression correlation Click here for file

Additional file 13 Mouse GO modules tissue expression difference test Click here for file
