# Supplementary figures and images for: Association of tissue lineage and gene expression: conservatively and differentially expressed genes define common and special functions of tissues
Source: BMC Bioinformatics. 2010 Dec 14;11(Suppl 11):S1. doi: 10.1186/1471-2105-11-S11-S1 (PMC3024865; doi:10.1186/1471-2105-11-S11-S1)

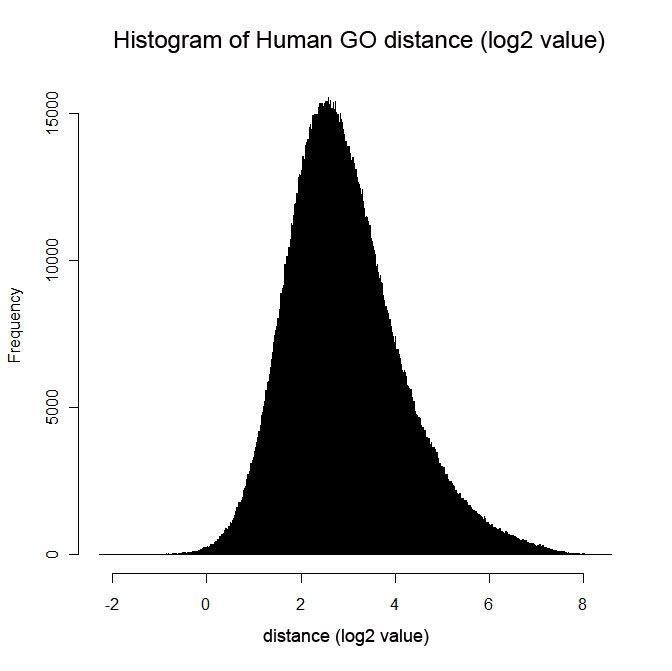

Supplement: Additional file 2 — Histogram of Human GO distance [file 1471-2105-11-S11-S1-S2.jpg]

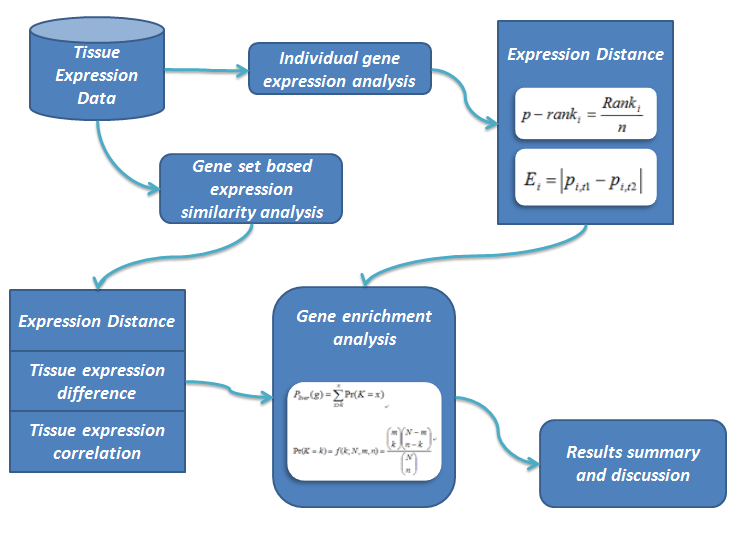

Supplement: Additional file 3 — Analysis workflow [file 1471-2105-11-S11-S1-S3.png]
